# Supplementary material for: Integrative analysis of the transcriptome and metabolome provides insights into polysaccharide accumulation in Polygonatum odoratum (Mill.) Druce rhizome
Source: PeerJ. 2024 Jul 9;12:e17699. doi: 10.7717/peerj.17699 (PMC11243984; doi:10.7717/peerj.17699)
Supplement: Supplemental Information 3 [file peerj-12-17699-s003.doc]

| **EXPERIMENTAL DESIGN** |  |  |  |
| --- | --- | --- | --- |
| Definition of experimental and control groups | **E** | This study was performed on the cDNA of rhizome of two varieties Y10 and Y11 | Line 13-14, Page 1 |
| Number within each group | **E** | At least two biological replicates | Line 143, Page 5 |
| Assay carried out by core lab or investigator's lab? | D |  |  |
| Acknowledgement of authors' contributions | D |  |  |
| **SAMPLE** |  |  |  |
| Description | **E** | The rhizomes of Polygonatum odoratum were collected in 10 mL tubes and kept in liquid nitrogen outdoor, and stored at −80 ℃ in the refrigerator in lab. | Line 103, page 4 |
| Volume/mass of sample processed | D |  |  |
| Microdissection or macrodissection | **E** | Not applicable |  |
| Processing procedure | **E** | The rhizomes were collected in 10 mL tubes using knife, and washed with sterile distilled water, then the rhizomes were immediately frozen in liquid nitrogen | Line 101-103, page 4 |
| If frozen - how and how quickly? | **E** | After washing with sterile distilled water, the rhizomes were immediately frozen in liquid nitrogen and stored at −80 ℃ | Line 101-103, page 4 |
| If fixed - with what, how quickly? | **E** | Not applicable |  |
| Sample storage conditions and duration (especially for FFPE samples) | **E** | stored at −80 ℃ into freezer; about 3 months | N/A |
| **NUCLEIC ACID EXTRACTION** |  |  |  |
| Procedure and/or instrumentation | **E** | (1) Take 500μl of lysate RLT, transfer it into a 1.5ml centrifuge tube, add 50μL of PLANTaid and mix well.  (2) Grind an appropriate amount of plant tissue into fine powder in liquid nitrogen, take 50mg-100mg of fine powder and transfer it into the above centrifuge tube with RLT and PLANTaid, immediately shake vigorously by hand for 20 seconds.  (3) Centrifuge the lysate at 13,000 rpm for 5-10 minutes, take the supernatant of the lysate and transfer it to a new centrifuge tube, add half of the volume of anhydrous ethanol to the supernatant.  (4) Add the above mixture to a genomic removal column and centrifuge at 13,000 rpm for 2 minutes, discard the waste liquid. Place the genomic DNA removal column in a clean 2 ml centrifuge tube then add 500 μL of lysate RLT Plus, centrifuge at 13,000 rpm for 30 seconds, collect the filtrate, and add half the volume of filtrate in anhydrous ethanol.  (5) Immediately add the mixture to an adsorbent column RA and centrifuge at 13,000 rpm for 2 min and discard the waste solution.  (6) Add 700 μL of deproteinising solution RW1, leave at room temperature for 1 min, centrifuge at 13,000 rpm for 30 s and discard the waste solution.  (7) Add 500 μL of rinse solution RW, centrifuge at 13,000 rpm for 30 seconds, and discard the waste solution. Add 500 μL of rinse solution RW and repeat.  (8) Place the adsorbent column RA back into the empty collection tube and centrifuge at 13,000 rpm for 2 min, then remove the adsorbent column RA and place it into an RNase free centrifuge tube, add 30-50 μL of RNase free water to the middle of the adsorbent membrane, leave it at room temperature for 1 min and then centrifuge at 12,000 rpm for 1 min. | N/A |
| Name of kit and details of any modifications | **E** | EASYspin Plus Plant RNA Kit (Aidlab Biotechnologies Co., Ltd., Beijing, China) | Line 110-111, Page 4 |
| Source of additional reagents used | D |  |  |
| Details of DNase or RNAse treatment | **E** | Add 50 µl of DNase-protected reaction solution to the centre of the membrane of the RNA mini Column and leave it at room temperature for 15 minutes. | N/A |
| Contamination assessment (DNA or RNA) | **E** | The quality and quantity of the total RNA were assessed on agarose gels and determined using a NanoDrop 2000 instrument (Thermo Fisher Scientific, Waltham, MA, USA) | Line 112-113, Page 4 |
| Nucleic acid quantification | **E** | determined using a NanoDrop 2000 instrument (Thermo Fisher Scientific, Waltham, MA, USA) | Line 112, Page 4 |
| Instrument and method | **E** | According to the manufacturer’s instructions of a NanoDrop 2000 instrument (Thermo Fisher Scientific, Waltham, MA, USA) | N/A |
| Purity (A260/A280) | D |  |  |
| Yield | D |  |  |
| RNA integrity method/instrument | **E** | Integrity RNA will usually have three bands detected by agarose gels, with the brightest band being the 28S band, followed by the 18S band, and the lightest band being the 5S band; the 28S band should be about twice as bright as the 18S band. | N/A |
| RIN/RQI or Cq of 3' and 5' transcripts | **E** | Not applicable |  |
| Electrophoresis traces | D |  |  |
| Inhibition testing (Cq dilutions, spike or other) | **E** | Not applicable |  |
| **REVERSE TRANSCRIPTION** |  |  |  |
| Complete reaction conditions | **E** | according to the instructions of the Evo M-MLV RT Premix for qPCR Kit (Accurate, Changsha, China) | Line 139=140, Page 5 |
| Amount of RNA and reaction volume | **E** | 1μg total RNA, and 20 μL reaction volume | N/A |
| Priming oligonucleotide (if using GSP) and concentration | **E** | Oligo (dt)17, 50μM | N/A |
| Reverse transcriptase and concentration | **E** | The enzyme RT6, 200U/μL | N/A |
| Temperature and time | **E** | 25 ℃ for 10 min, followed by 55 ℃ for 15min, and 85℃ for 5 min |  |
| Manufacturer of reagents and catalogue numbers | D |  |  |
| Cqs with and without RT | D* |  |  |
| Storage conditions of cDNA | D |  |  |
| **qPCR TARGET INFORMATION** |  |  |  |
| If multiplex, efficiency and LOD of each assay. | **E** | Not applicable |  |
| Sequence accession number | **E** | Not applicable (Because the genome of Polygonatum odoratum have not been sequenced) | N/A |
| Location of amplicon | D |  |  |
| Amplicon length | **E** | range from 80 bp to 250 bp | N/A |
| In silico specificity screen (BLAST, etc) | **E** | blast with the transcritome data | N/A |
| Pseudogenes, retropseudogenes or other homologs? | D |  |  |
| Sequence alignment | D |  |  |
| Secondary structure analysis of amplicon | D |  |  |
| Location of each primer by exon or intron (if applicable) | **E** | Not applicable |  |
| What splice variants are targeted? | **E** | Not applicable (Because the genome of Polygonatum odoratum have not been sequenced，we do not known how many variants the target gene has) |  |
| **qPCR OLIGONUCLEOTIDES** |  |  |  |
| Primer sequences | **E** | listed in Figure S2 | Figure S2 |
| RTPrimerDB Identification Number | D |  |  |
| Probe sequences | D** |  |  |
| Location and identity of any modifications | **E** | Not applicable |  |
| Manufacturer of oligonucleotides | D |  |  |
| Purification method | D |  |  |
| **qPCR PROTOCOL** |  |  |  |
| Complete reaction conditions | **E** | according to the instructions of the SYBR® Green Premix Pro Taq HS qPCR Kit (Accurate, Changsha, China) | Line 140, Page 5 |
| Reaction volume and amount of cDNA/DNA | **E** | RNA: 4 μL ; Reaction volume: 20 μL | N/A |
| Primer, (probe), Mg++ and dNTP concentrations | **E** | Primers: 200 nM; Mg++ and dNTP concentrations:NA, because this kit contained a mix buffer including SYBR green I, Mg++ and dNTP, and do not have detailed information about the concentration, It could be because it's a trade secret. | N/A |
| Polymerase identity and concentration | **E** | Not applicable |  |
| Buffer/kit identity and manufacturer | **E** | the SYBR® Green Premix Pro Taq HS qPCR Kit (Accurate, Changsha, China) | Line 140, Page 5 |
| Exact chemical constitution of the buffer | D |  |  |
| Additives (SYBR Green I, DMSO, etc.) | **E** | SYBR green I | Line 140, Page 5 |
| Manufacturer of plates/tubes and catalog number | D |  |  |
| Complete thermocycling parameters | **E** | 95 ℃ for 30 sec, followed by 40 cycles with 95 ℃ for 5 sec, and 60℃ for 30 sec | N/A |
| Reaction setup (manual/robotic) | D |  |  |
| Manufacturer of qPCR instrument | **E** | a 7500 Sequence Detection System (Applied Biosystems, USA) | N/A |
| **qPCR VALIDATION** |  |  |  |
| Evidence of optimisation (from gradients) | D |  |  |
| Specificity (gel, sequence, melt, or digest) | **E** | showed no significant homologies with the transcriptome data, and the dissociation curve of gene only has one peak | N/A |
| For SYBR Green I, Cq of the NTC | **E** | SYBR Green I |  |
| Standard curves with slope and y-intercept | **E** | NA |  |
| PCR efficiency calculated from slope | **E** | NA |  |
| Confidence interval for PCR efficiency or standard error | D |  |  |
| r2 of standard curve | **E** | NA |  |
| Linear dynamic range | **E** | LOD was identified by extracting and testing 10-fold serial dilutions of characterized stocks of in vitro transcribed RNA. | N/A |
| Cq variation at lower limit | **E** | 0.5-1.3 | N/A |
| Confidence intervals throughout range | D |  |  |
| Evidence for limit of detection | **E** | 100.5-100 copies. | N/A |
| If multiplex, efficiency and LOD of each assay. | **E** | NA |  |
| **DATA ANALYSIS** |  |  |  |
| qPCR analysis program (source, version) | **E** | QuantStudio Design & Analysis Software v1.5.1 (Applied Biosystems). | N/A |
| Cq method determination | **E** | Standard curve. | N/A |
| Outlier identification and disposition | **E** | NA |  |
| Results of NTCs | **E** | Negative | N/A |
| Justification of number and choice of reference genes | **E** | NA |  |
| Description of normalisation method | **E** | Passive reference (ROX) | N/A |
| Number and concordance of biological replicates | D |  |  |
| Number and stage (RT or qPCR) of technical replicates | **E** | at least 2 technical replicates | Line 143, Page 5 |
| Repeatability (intra-assay variation) | E | 98% | N/A |
| Reproducibility (inter-assay variation, %CV) | D |  |  |
| Power analysis | D |  |  |
| Statistical methods for result significance | **E** | one-way ANOVA | N/A |
| Software (source, version) | E | IBM SPSS version 26 | N/A |
| Cq or raw data submission using RDML | **D** |  |  |
