# Supplementary figures and images for: Integrative analysis of the transcriptome and metabolome provides insights into polysaccharide accumulation in Polygonatum odoratum (Mill.) Druce rhizome
Source: PeerJ. 2024 Jul 9;12:e17699. doi: 10.7717/peerj.17699 (PMC11243984; doi:10.7717/peerj.17699)

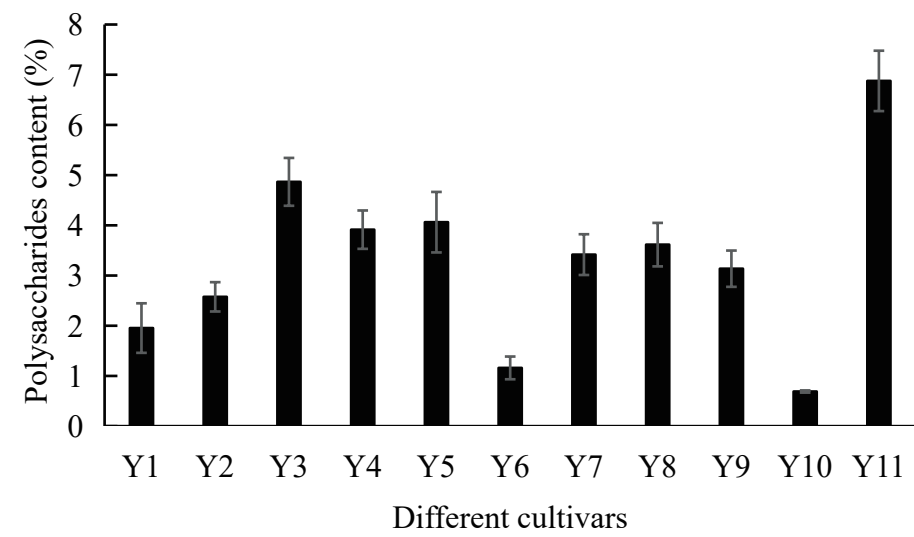

Supplement: Supplemental Information 4 [file peerj-12-17699-s004.pdf]

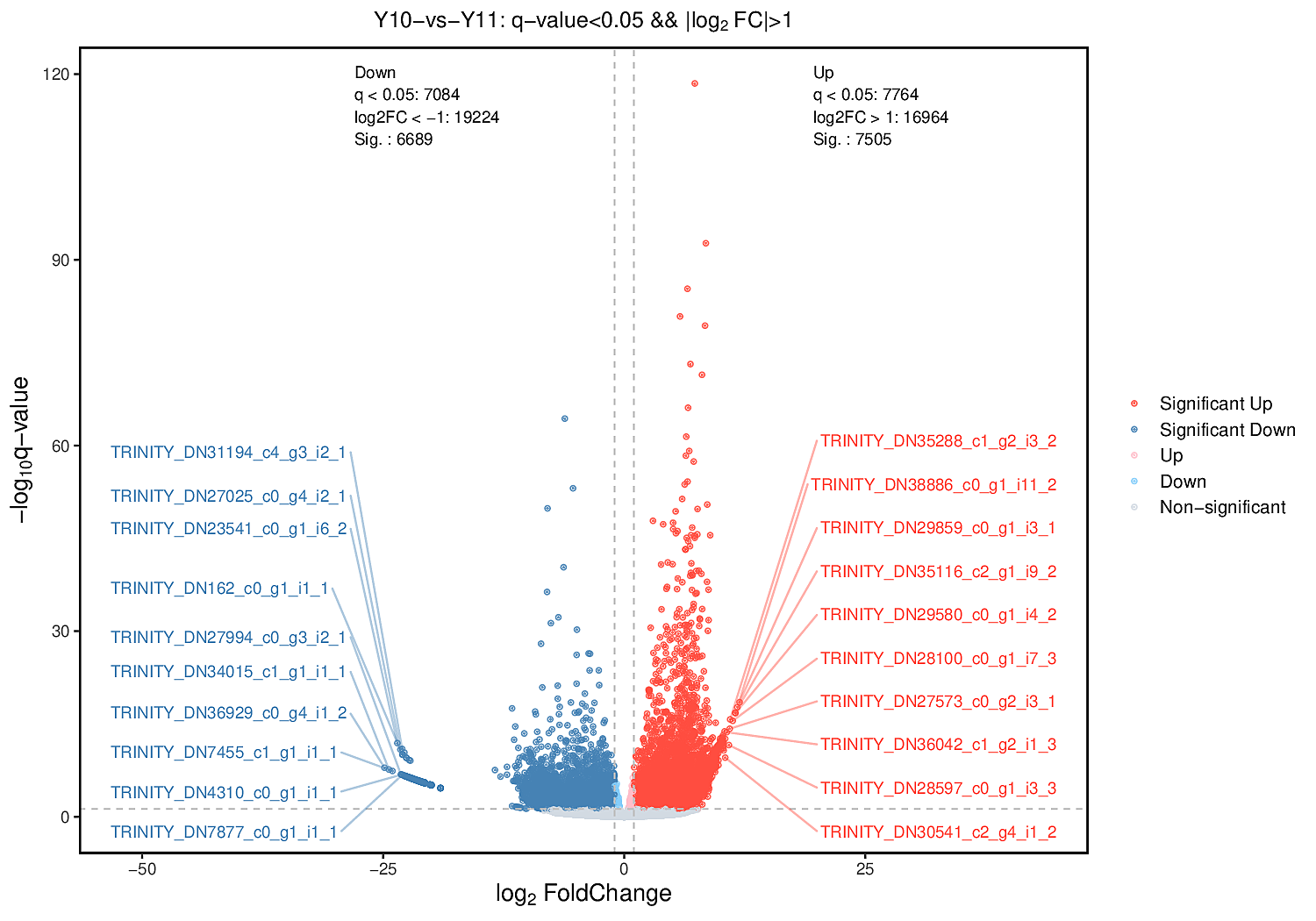

Supplement: Supplemental Information 5 [file peerj-12-17699-s005.png]

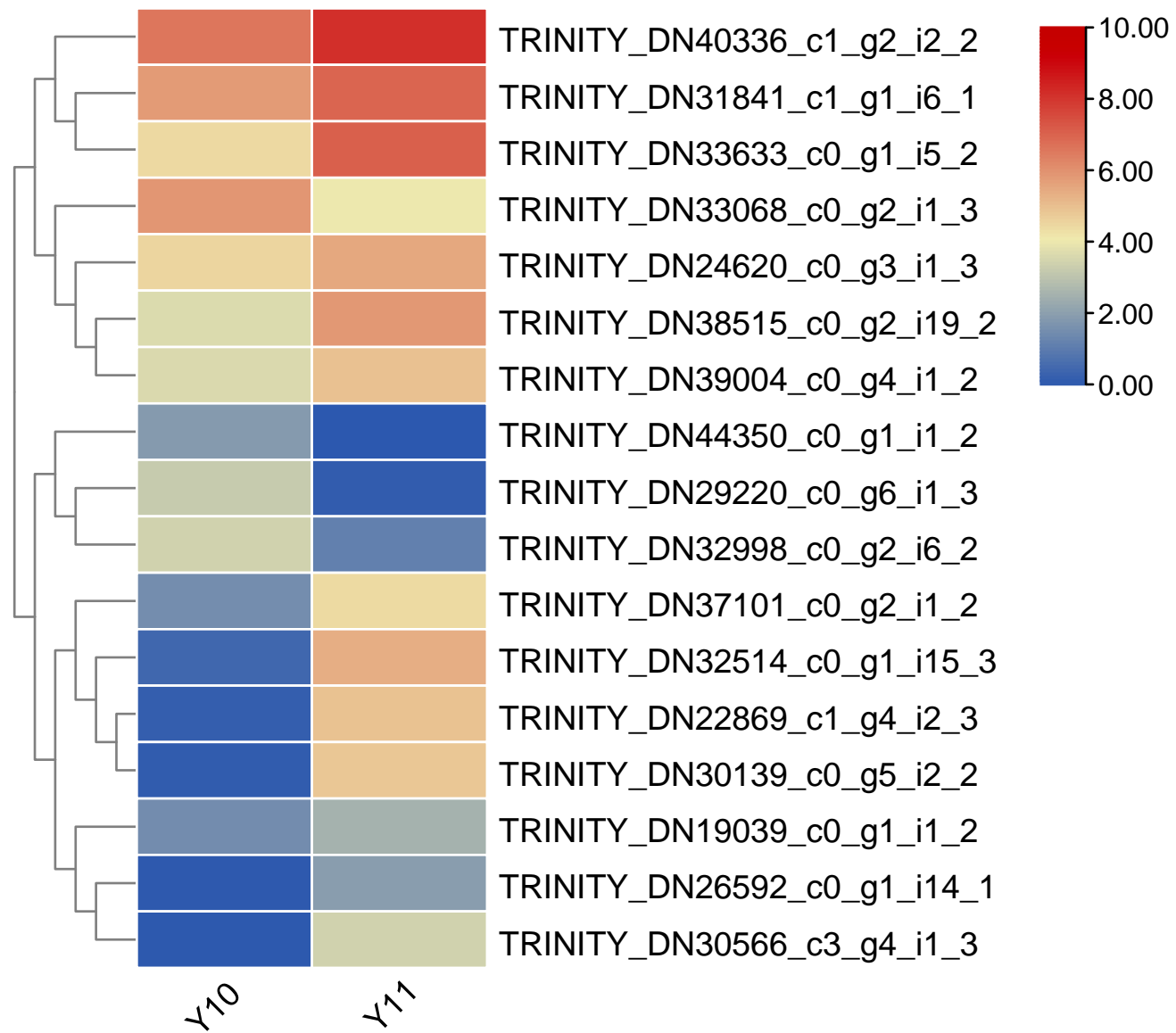

Supplement: Supplemental Information 6 [file peerj-12-17699-s006.pdf]

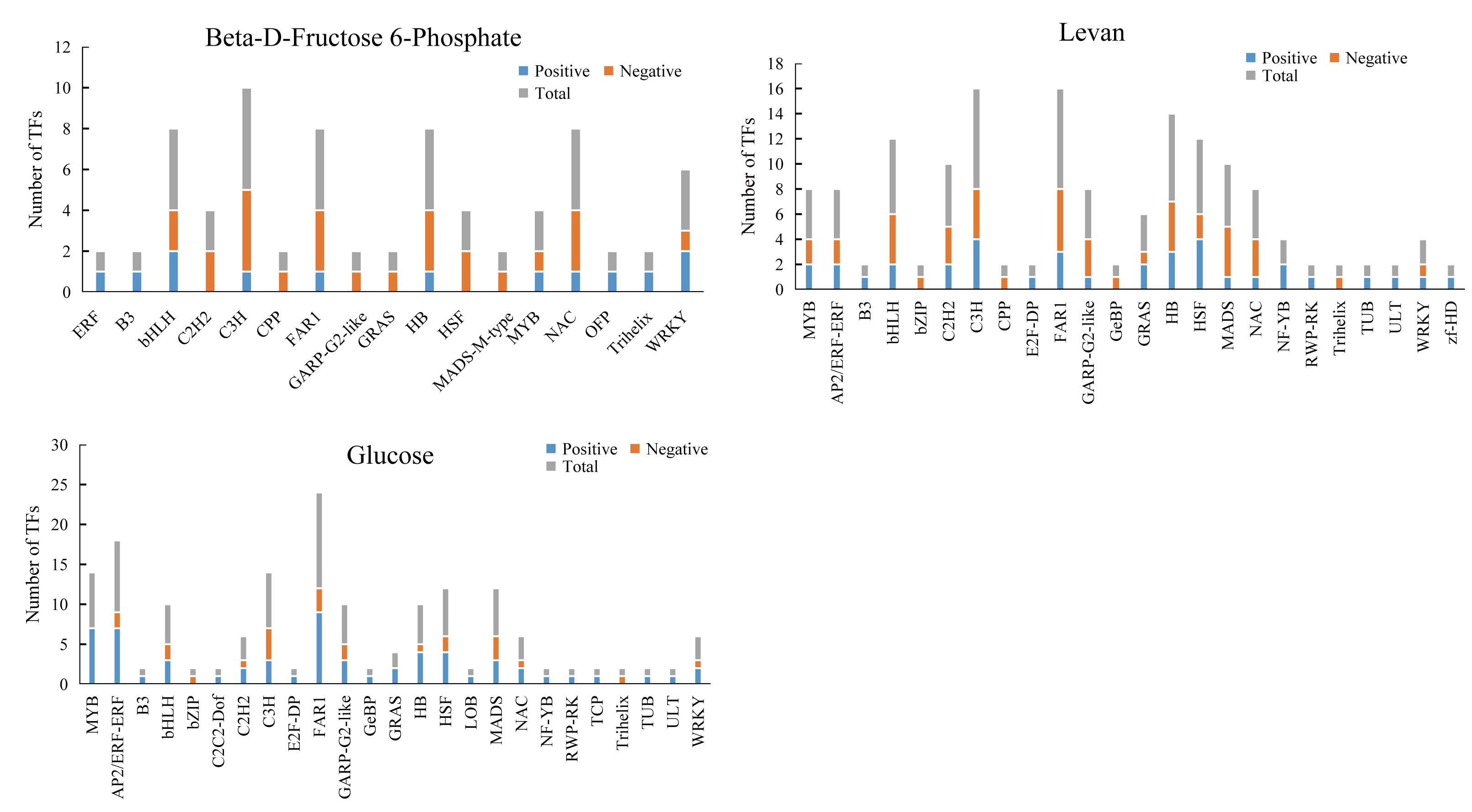

Supplement: Supplemental Information 7 [file peerj-12-17699-s007.png]

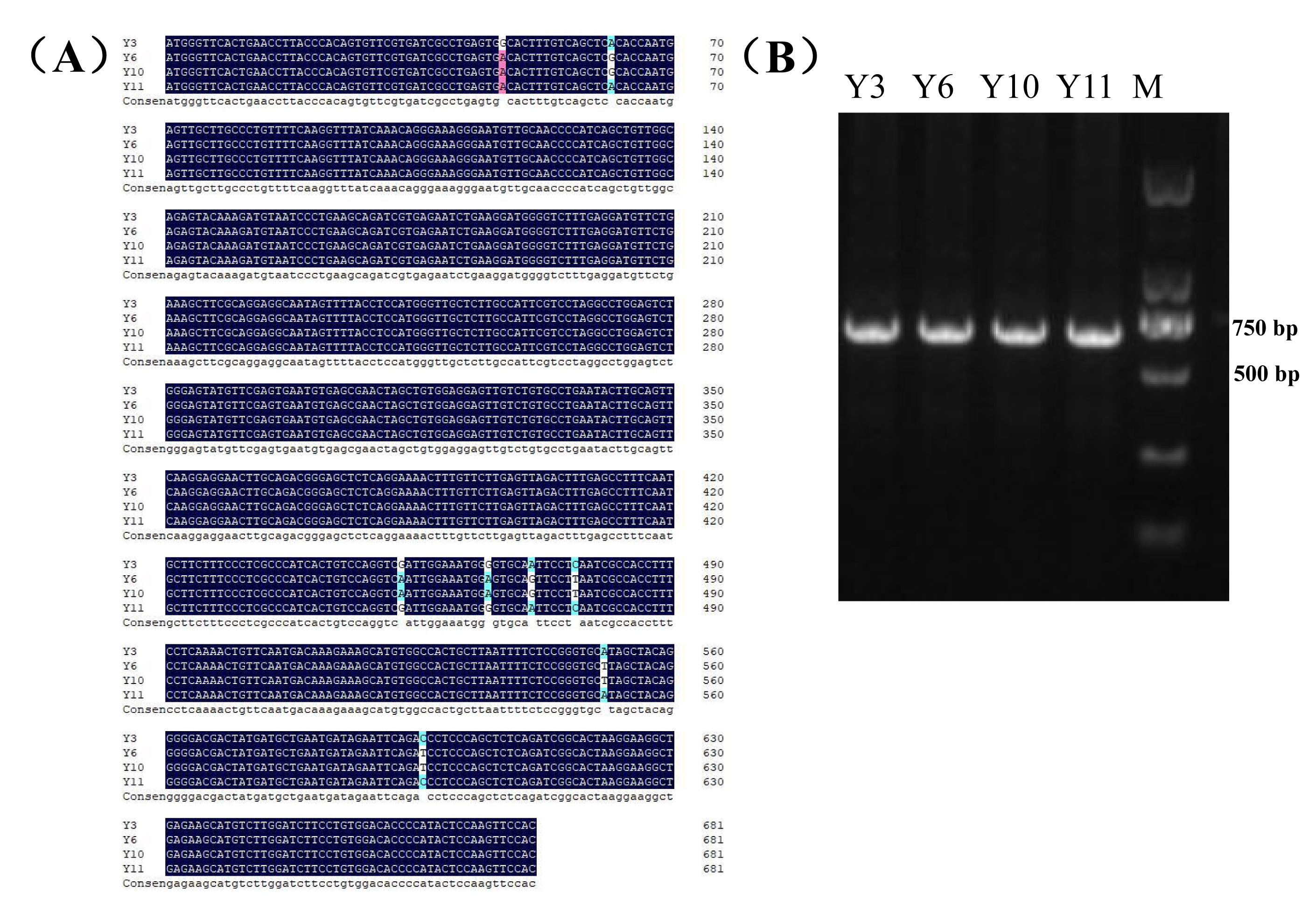

Supplement: Supplemental Information 8 [file peerj-12-17699-s008.jpg]
