# Supplementary material for: Integrative analysis of the transcriptome and metabolome provides insights into polysaccharide accumulation in Polygonatum odoratum (Mill.) Druce rhizome
Source: PeerJ. 2024 Jul 9;12:e17699. doi: 10.7717/peerj.17699 (PMC11243984; doi:10.7717/peerj.17699)
Supplement: Supplemental Information 9 [file peerj-12-17699-s009.doc]

Table S1 The basic information of transcriptom of rhizome in Y11 and Y10

| Sample | RawReads(M) | RawBases(G) | CleanReads(M) | CleanBases(G) | ValidBases(%) | Q30(%) | GC(%) |
| --- | --- | --- | --- | --- | --- | --- | --- |
| Y10-1 | 50.34 | 7.55 | 50.27 | 7.4 | 98.04 | 93.39 | 48.07 |
| Y10-2 | 46.58 | 6.99 | 46.51 | 6.87 | 98.26 | 92.33 | 47.86 |
| Y10-3 | 49.3 | 7.39 | 49.23 | 7.28 | 98.44 | 92.72 | 47.88 |
| Y11-1 | 47.53 | 7.13 | 47.46 | 6.98 | 97.95 | 93.6 | 47.99 |
| Y11-2 | 50.5 | 7.57 | 50.44 | 7.47 | 98.55 | 92.56 | 47.67 |
| Y11-3 | 47.33 | 7.1 | 47.27 | 7 | 98.6 | 93.2 | 47.33 |
