# Supplementary material for: Integrative analysis of the transcriptome and metabolome provides insights into polysaccharide accumulation in Polygonatum odoratum (Mill.) Druce rhizome
Source: PeerJ. 2024 Jul 9;12:e17699. doi: 10.7717/peerj.17699 (PMC11243984; doi:10.7717/peerj.17699)
Supplement: Supplemental Information 10 [file peerj-12-17699-s010.doc]

Table S2 The primers used in this study

| Name | Primer | Primer sequence (5’-3’) | Application |
| --- | --- | --- | --- |
| TRINITY_DN21530_c0_g1_i1_1 | forward | AATGCTCCAAGCCATTCGTC | Quantitative Real-time PCR |
|  | reverse | CCAACAAACACACAGAGGCA |  |
| TRINITY_DN29047_c1_g1_i18_3 | forward | CAAGCTTGGAGAGGGAGTGA |  |
|  | reverse | TTTGTGTAAGCACGGAGCAG |  |
| TRINITY_DN31666_c1_g1_i9_3 | forward | CTTGCCAGGAAGGCCATAAC |  |
|  | reverse | AAGCCATCTCAGGGTTCTCC |  |
| TRINITY_DN34969_c0_g1_i3_2 | forward | CTTGCCAGGAAGGCCATAAC |  |
|  | reverse | AAGCCATCTCAGGGTTCTCC |  |
| TRINITY_DN29714_c0_g1_i1_3 | forward | GCAGGAACTGGAAGTGTGTC |  |
|  | reverse | ACAGTCCTACGGCTGAACAA |  |
| TRINITY_DN24830_c0_g1_i4_1 | forward | AGCATTTGCAGGGTTCAGTG |  |
|  | reverse | ACCTCGGCGATGTAATGACT |  |
| TRINITY_DN33146_c0_g1_i3_3 | forward | TCCAATCACCCTCCAAGACC |  |
|  | reverse | GAAGAAGCCAGGGAAGGAGT |  |
| TRINITY_DN33229_c0_g1_i4_3 | forward | TACCATTTGGCTACCGTGGA |  |
|  | reverse | GTGTGCCATTTCCACCAAGT |  |
| TRINITY_DN33229_c0_g2_i1_3 | forward | GGCATATGCAACACGCACTA |  |
|  | reverse | TGGCCTGTAGATGTCAAGCA |  |
| TRINITY_DN26592_c0_g1_i14_1 (HK) | forward | ATGCTACTCTCGCGAACAGA |  |
|  | reverse | CTGGACCGCATACTTTCAGC |  |
| TRINITY_DN44350_c0_g1_i1_2 (UGDH) | forward | TCCTCCTTCCTCTGTTGCAG |  |
|  | reverse | CCCTGGCCTTCACCTAAAGA |  |
| TRINITY_DN40239_c1_g3_i1_2 (SUS) | forward | TCGTGAAACTAAGCGCTACC |  |
|  | reverse | AACAGTAAATTGGATCATTGGC |  |
| TRINITY_DN25011_c0_g2_i7_1 (UGP2) | forward | CGATCAGATCAGCGAGAACG |  |
|  | reverse | TGCGCTTCTCCACTCAAGTA |  |
| actin | forward | AAGTTGCTGGAATCCACGAG | Reference gene |
|  | reverse | CTCATACGATCAGCAATACC |  |
